# Unusual Presentation and Difficult to Diagnose: A Case of Malaria With Negative Thick and Thin Giemsa Stain Smear Tests

**DOI:** 10.7759/cureus.39675

**Published:** 2023-05-29

**Authors:** Oshna Pandey, Elisha Hona, Elina Shrestha, Varsha Khadka, Tsewang Ghising

**Affiliations:** 1 School of Medicine, Patan Academy of Health Sciences, Kathmandu, NPL; 2 Internal Medicine, Nepal Korea Friendship Municipality Hospital, Bhaktapur, NPL; 3 Internal Medicine, BronxCare Health System, Bronx, USA

**Keywords:** ascites, pleural effusion, rt-pcr, negative giemsa, malaria

## Abstract

Malaria is a parasitic disease that is spread by the bite of an Anopheles mosquito carrying the infection. Microscopic analysis of thick and thin Giemsa-stained smears is the gold standard for diagnosis. If the initial test is negative, but clinical suspicion is high, further smears are required. A 25-year-old man presented with abdominal distension, cough, and a seven-day fever. In addition, the patient developed pleural effusions and ascites. The thick and thin smear tests for malaria and all other fever testing came out negative. Plasmodium vivax was later identified by reverse transcription polymerase chain reaction (RT-PCR). There was a considerable improvement once the anti-malarial medicine was started. It was difficult to diagnose him because pleural effusion and ascites are unusual for someone with malaria. Furthermore, several Giemsa stain smears and malaria quick diagnostic tests were negative, and only a few labs in our country performed RT-PCR.

## Introduction

Malaria is a parasitic disease transmitted via the bite of an infected Anopheles mosquito that may lead to life-threatening illness [1]. It continues to be a major cause of morbidity and mortality in developing countries. The disease can present with cyclic fever with chills, headache, malaise, gastrointestinal distress, upper respiratory symptoms, jaundice, anemia, and hepatosplenomegaly; severe cases may include hepatic failure, renal failure, seizure, and altered sensorium [2]. However, some cases could exhibit atypical signs and symptoms, making it challenging to diagnose and treat them promptly.

The gold standard for malaria diagnosis is a microscopic examination of Giemsa-stained thick and thin smears. To avoid low-level parasitemia, examinations must be performed with oil immersion at 100 and 1000 times magnification [3]. A negative smear does not rule out malaria, and if clinical suspicion is high and microscopy is negative, smears must be repeated in 12 and 24 hours. Polymerase chain reaction (PCR) is used to diagnose low-level parasitemia. Here, we present a case of thick and thin smear-negative malaria with polyserositis (pleural effusion and ascites) [4].

## Case presentation

A 25-year-old male presented to the emergency department with the chief complaint of fever for seven days, cough with occasional whitish sputum, and a distended abdomen for three days. The maximum recorded temperature was 102 °F with an evening rise and was not associated with chills or rigor. He had no prior history of malaria or travel history to malaria-endemic areas.

Initially, the patient was treated at a nearby medical clinic with a combination of azithromycin 500 mg for five days and cefixime 200 mg for seven days, as well as antipyretics and cough suppressants. As his symptoms were persistent, he visited our institution for further management.

On presentation, the patient looked ill, conscious, and oriented to time, place, and person, with a temperature of 102 °F, a pulse of 110 beats per minute (bpm), a blood pressure of 110/80 mmHg, a respiratory rate of 24 breaths per minute, and a SpO2 of 96% at room air. On examination, there was no pallor, icterus, or pedal oedema, but there was decreased air entry over the right lower hemithorax with bilateral basilar crepitation. The abdominal examination was positive for a fluid thrill. There was no significant lymphadenopathy, and the rest of the examination was unremarkable.

Investigation (Table 1) revealed a TLC of 7800/cumm with 70% neutrophils and 24% lymphocytes and a hemoglobin level of 12.2 g/dl. A peripheral blood smear revealed no abnormal cells, with RBC: normocytic and normochromic, WBC: within normal limits (neutrophils 70%, lymphocytes 20%, monocytes 6%, and eosinophils 4%), and platelets: normal. In the first hour, the erythrocyte sedimentation rate was 22 mm and the C-reactive protein was 183 mg/l. The liver function tests (test bilirubin: 0.4 mg/dl, direct bilirubin: 0.2 mg/dl, aspartate aminotransferase (AST): 15.7 U/L, alanine aminotransferase (ALT): 17.4 U/ml, alkaline phosphatase: 70 U/L) and renal function tests (blood urea: 24 mg/dl, serum creatinine: 1 mg/dl, serum sodium: 138 mg/dl, serum potassium: 4.2 mg/dl) were within the reference range.

**Table 1:** Laboratory studies of the patient. SGOT: serum glutamic-oxaloacetic transaminase; AST: aspartate aminotransferase; SGPT: serum glutamate pyruvate transaminase; ALT: alanine aminotransferase; ADA: adenosine deaminase cell/mm^3^: cells per milliliter; gm/dl: gram per deciliter; mg/dl: milligram per deciliter; mEq/L: milliequivalent per liter; IU/L: international units per liter

Laboratory parameters	Values	Reference range
Total leucocyte count	7800	4000-11000 cells/mm^3 ^
Neutrophils	70	40%-75%
Lymphocytes	24	20%-50%
Eosinophils	2	1%-6%
Monocytes	4	2%-10%
Basophils		0%-1%
Hemoglobin	12.2	13-18 g/dl
Platelets	248000	150000-400000 cells/mm^3^
Urea	24	15-45 mg/dl
Creatinine	1	0.4-1.4 mg/dl
Sodium	138	135-146 mEq/L
Potassium	4.2	3.5-5.3 mEq/L
Total Bilirubin	0.4	0.4-1 mg/dl
Direct Bilirubin	0.2	0-0.4 mg/dl
SGOT/AST	15.7	<37 U/L
SGPT/ALT	17.4	5-35 U/ml
Alkaline Phosphatase	70	53-128 U/L
Serum Protein	2.86	3-5 g/dl
Serum Lactate Dehydrogenase (LDH)	349	225-450 U/L
Pleural Fluid Protein	3.89	6-8 g/dl
Pleural Fluid LDH	387	
Pleural Fluid ADA	13	0-40 IU/L
Ascitic Fluid ADA	16	0-40 IU/L

**Figure 1:**
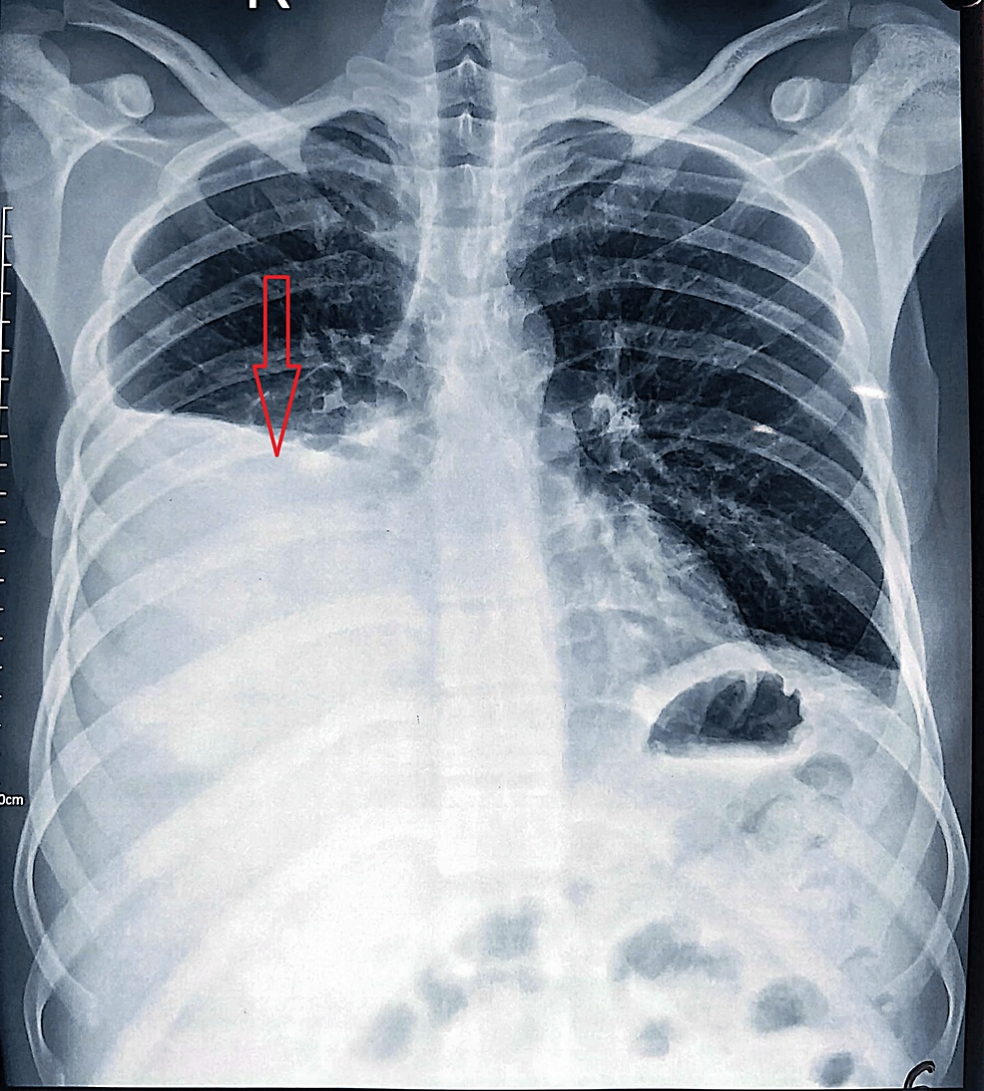
Chest X-ray showing right-sided pleural effusion

Chest X-ray (Figure 1) showed right-sided pleural effusion. Sputum samples were negative for Gene Xpert testing, and acid-fast bacillus (AFB) smears for three consecutive days were negative. Serological tests for the fever panel (scrub typhus, brucellosis, leptospirosis, malaria, and dengue) were all negative. Microscopic evaluation of Giemsa-stained thick and thin smears for malaria was negative.

An initial ultrasonogram of the abdomen and pelvis revealed moderate right and mild left pleural effusions and gross ascites. Diagnostic and therapeutic ascitic and pleural tapping was done.

The serum ascites albumin gradient (SAAG) was found to be 0.5 mg/dl (low albumin gradient), indicating an exudative etiology. Pleural tapping, according to Light's criteria, was likewise indicative of an exudative cause (Table 1).

We also found gross ascites with diffuse omental thickening and nodularity, a thickened and enhancing peritoneal fold, a mild thickening and enhancing wall of the terminal ileum, a few enhancing necrotic mesenteric lymph nodes, moderate right and mild left pleural effusions, partial collapse/consolidation of the right middle lobe, and enlarged subcarinal and hepatic hilum lymph nodes. However, the possibility of underlying metastatic disease must be ruled out.

On the basis of the above results, although there was no evidence of *Mycobacterium tuberculosis*, anti-tubercular therapy was started with dosing as per directly-observed therapy, short-course. Despite starting anti-tuberculosis therapy, the patient didn't improve clinically. Ascitic and pleural tapping were repeated for therapeutic and diagnostic purposes. Cytology and cell block for malignant cells, AFB and gene Xpert, Gram stain and culture, and fever panel serology (scrub typhus, brucellosis, leptospirosis, malaria, and dengue) were performed on both fluid samples. All the reports were unremarkable.

**Figure 2:**
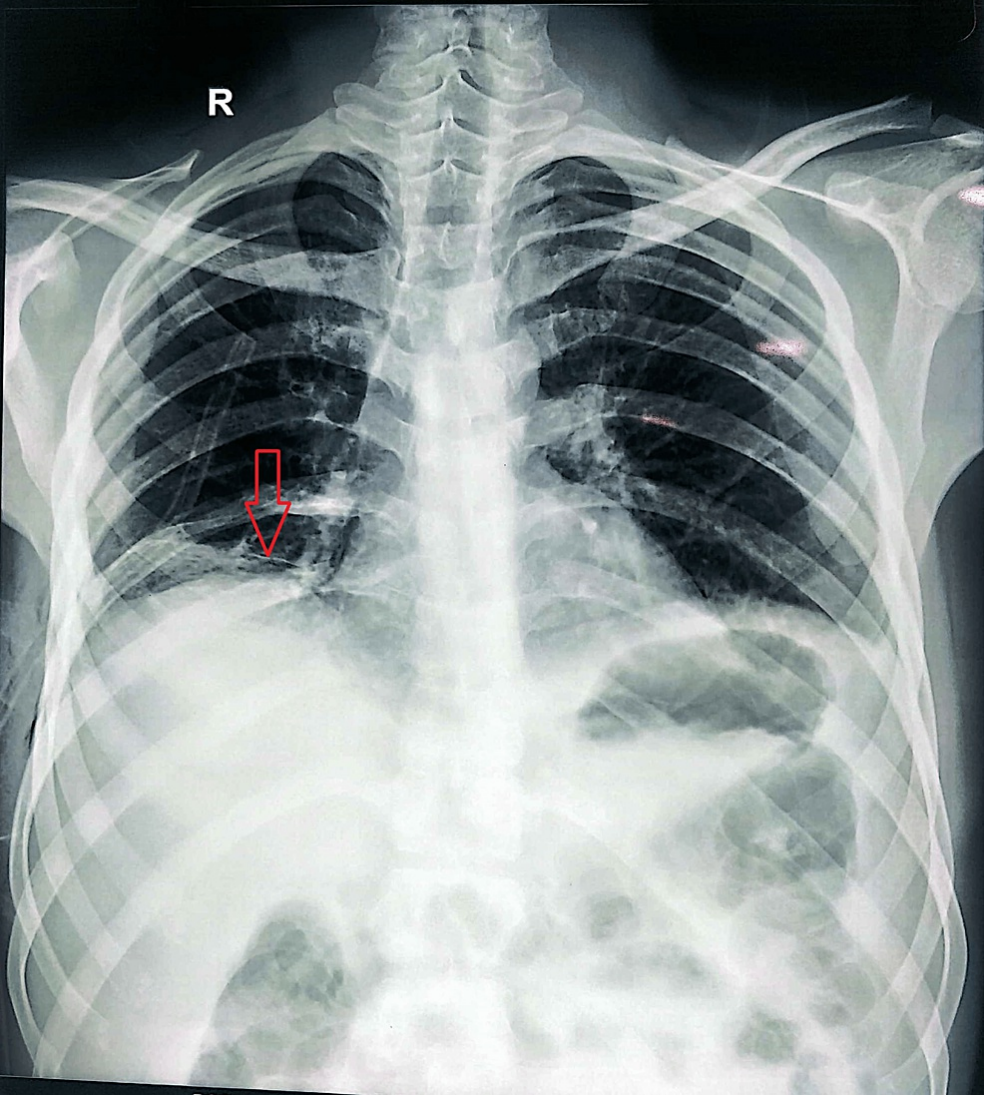
Chest X-ray post-chest-tube insertion

A sample was sent to the central lab for RT-PCR for malaria, and it tested positive for *Plasmodium vivax*. The patient was given antimalarial medication (a combination of artemether and lumefantrine). Surprisingly, his fever began to subside after 72 hours. Clinically, the patient improved significantly. Gradually, his ascites and pleural effusion subsided. He was discharged after the chest tube drain was removed (Figure 2). Subsequently, he was having regular check-ups and was doing well.

## Discussion

The most common clinical features of malaria include fever with chills and rigor, headache, joint pain, and fatigue [2]. The two most significant complications of malaria are cerebral malaria and nephrotic syndrome. However, this case presented with unusual features, namely, fever with polyserositis (pleural effusion and ascites), which made it difficult to make a diagnosis.

The gold standard for malaria diagnosis remains microscopic examination of Giemsa-stained thick and thin smears. To avoid low-level parasitemia, examinations are performed at 100 and 1000 times magnification with oil immersion. A negative smear does not rule out malaria, and if clinical suspicion is high and microscopy is negative, smears must be repeated in 12 and 24 hours. Similarly, in our cases, we sent smears for Giemsa stain multiple times; however, they all came back negative. Moreover, the patient was started on antitubercular therapy on an experimental basis. In our country, only a few labs perform RT-PCR for malaria in non-endemic zones. As the patient was not improving clinically and the fever was persistent, our team decided to send RT-PCR for malaria to the central lab, the only lab in our district with a PCR facility for malaria. We were working diligently to get a diagnosis in every way possible. Finally, we sent a malaria RT-PCR sample to a central lab, and the sample tested positive for *Plasmodium vivax*. As demonstrated in our case, the PCR test is useful for low-level parasitemia. In a case series of 17 patients published by Alexandre et al. [5], *Plasmodium vivax* malaria, once thought to be a benign disease, is now causing increasingly severe malaria [6]. It has been demonstrated that it has the potential to cause multiorgan dysfunction syndrome [3].

Severe malaria is a life-threatening but very treatable disease. Nonspecific and uncommon clinical symptoms [4], as in our case (ascites, pleural effusions, fever), may lead treating physicians who see malaria infrequently to have a delay in diagnosis, thus leading to a delay in treatment [2]. Therefore, it is very important to investigate thoroughly and initiate appropriate treatment to prevent morbidity and mortality.

## Conclusions

Although fever is the principal symptom of malaria, its clinical symptoms can vary and are sometimes unusual, which makes it difficult to make a timely diagnosis and treat it appropriately. Microscopic evaluation of Giemsa-stained thick and thin smears remains the gold standard for malaria diagnosis; however, the results could be negative in the initial phase. If clinical suspicion is high, PCR can be a useful modality for the early diagnosis of patients with suspected malaria and patients with low-level parasitemia.

## References

[1] Bartoloni A, Zammarchi L (2012). Clinical aspects of uncomplicated and severe malaria. Mediterr J Hematol Infect Dis.

[2] Goyal VK, Chhangani NP, Jora R, Sundararajan S (2015). Vivax malaria infection manifesting as fulminant hepatic failure: a case report. Int J Clin Pediatrics.

[3] Phillips MA, Burrows JN, Manyando C, van Huijsduijnen RH, Van Voorhis WC, Wells TN (2017). Malaria. Nat Rev Dis Primers.

[4] Zaki SA, Shanbag P (2011). Atypical manifestations of malaria. Res Rep Trop Med.

[5] Alexandre MA, Ferreira CO, Siqueira AM, Magalhães BL, Mourão MP, Lacerda MV, Alecrim Md (2010). Severe Plasmodium vivax malaria, Brazilian Amazon. Emerg Infect Dis.

[6] Douglas NM, Pontororing GJ, Lampah DA (2014). Mortality attributable to Plasmodium vivax malaria: a clinical audit from Papua, Indonesia. BMC Med.

